# Sepsis-Related Mortality of Very Low Birth Weight Brazilian Infants: The Role of *Pseudomonas aeruginosa*

**DOI:** 10.1155/2009/427682

**Published:** 2010-02-21

**Authors:** Sylvia Maria Porto Pereira, Maria Helena Cabral de Almeida Cardoso, Ana Lucia Figuexeds, Haroldo Mattos, Ronaldo Rozembaum, Vanessa Isidoro Ferreira, Maria Antonieta Portinho, Ana Cristina Gonçalves, Elaine Sobral da Costa

**Affiliations:** ^1^Post-Graduate Department, Fernandes Figueira Institute, Rui Barbosa Avenue, 716, Flamengo, Rio de Janeiro 22250-020, Brazil; ^2^Neonatology Department, Servidores do Estado Hospital, Sacadura Cabral Street 178, Saúde, Rio de Janeiro 20221-903, Brazil; ^3^Pediatrics Institute IPPMG Pediatrics Department, Federal University of Rio de Janeiro, Bruno Lobo Avenue, 50, Ilha do Fundão, Rio de Janeiro 21914 912, Brazil; ^4^Estacio De Sa University, Riachvelo Street 27-Centro, Riode Janeiro, CEP 20230-010, Brazil; ^5^Epidemiology Department, Servidores do Estado Hospital, Sacadura Cabral Street 178, Saúde, Rio de Janeiro 20221-903, Brazil; ^6^Hospital Infection Control Department, Servidores do Estado Hospital, Sacadura Cabral Street 178, Saúde, Rio de Janeiro 20221-903, Brazil; ^7^Medical Residence, Fernandes Figueira Institute, Rui Barbosa Avenue, 716, Flamengo, Rio de Janeiro 22250-020, Brazil

## Abstract

The aim of this study is to identify risk factors for sepsis-related mortality in low birth weight (<1500 g) infants. We performed retrospective cohort study to investigate risk factors for sepsis-related mortality in all neonates birth weight <1500 g admitted to Level III neonatal intensive care unit, Brazil, April 2001/September 2004. Of the 203 cases, 71 (35%) had sepsis. Of those, gram-positive was identified in 52/87 blood cultures (59.8%), the most common *Coagulase-negative Staphylococcus* (31/87; 35.5%). Gram-negative was present in 29 of the 87 positive blood cultures (33.3%), with *Pseudomonas aeruginosa* (8/87; 9.1%), the most frequent agent. Overall 21 of 71 infants with sepsis (29.6%) died. Risk factors for sepsis-related mortality were gestational age ≤28 weeks, birth weight ≤1000 g (9.6 times more often than birth weight >1000 g), five-minute Apgar ≤7, gram-negative sepsis, mechanical ventilation (6.7 times higher than no use), and intravascular catheter. Sepsis-related mortality was due, mainly, to *Pseudomonas aeruginosa*; birth weight ≤1000 g and mechanical ventilation were strong sepsis-related mortality predictors.

## 1. Introduction

Neonatal sepsis is a frequent complication of very low birth weight (VLBW) infants and it is an important cause of neonatal morbidity and mortality [1, 2]. VLBW infants develop 2.7 times more sepsis than other infants since their immune system and skin barrier are immature and they are exposed to many invasive diagnostic and therapeutic procedures [3]. Morbidity and mortality in a neonatal intensive care unit (NICU) can be reduced by knowledge of the epidemiology of the microbiology, infection rate profile and antibiotic sensitivity, and by introducing practices that are based on clinical evidence [4, 5]. Special attention must be given to infection by *Pseudomonas aeruginosa* due to a high mortality rate [6]. The aim of this study is to identify the risk factors involved in the mortality caused by sepsis of a VLBW infants population with sepsis, in particular, the role of *Pseudomonas aeruginosa*, a very aggressive pathogen. The infants were hospitalized in the NICU of a high-risk maternity in a tertiary-level public hospital specialized in caring for highly complex patients. Secondary objectives are to determine the frequency and distribution of the pathogens that cause infection and to describe the characteristics of this population.

## 2. Methods

### 2.1. Patients

All VLBW infants were born at the maternity of Servidores do Estado Hospital (SHE), Rio de Janeiro, RJ, Brazil and admitted in the NICU of the same hospital, just after birth, between 1 April, 2001 and 30 September, 2004 meeting the following criteria: birth weight (BW) <1500 g, clinical evidence of systemic infection and positive blood culture result for bacterium or yeast on one or more blood cultures obtained at any time while infants were inpatients in the NICU. Infants died of nonsepsis causes, with lethal congenital malformations and chromosomal abnormalities were excluded.

### 2.2. Chart Review

This retrospective cohort study collected data from the medical records of the selected VLBW infants using an investigation protocol. Relevant informations included *Antenatal and Intrapartum History*: gestational age (GA) based on the date of the last menstruation, uterus fundus measurement, and ultrasonography performed until 12 weeks of GA; time elapsed between rupture of membranes and birth; mode of birth; Apgar score at the first, fifth, and tenth minute of life; gender; BW; BW and GA relationship according to Usher and McLean's growth curves [7]. *Events Related to Infection Episodes*: incidence of sepsis, defined by the presence of clinical signs and symptoms as apnea, gastrointestinal problems, increased need for oxygen or ventilatory support, and lethargy/hypotonic, and by one or more positive blood cultures for bacterium or yeast obtained at any time during the infant's stay at the NICU. Any organism, including *Coagulase-negative Staphylococcus* (CONS) was considered the caused agent of sepsis and not a contaminant if the criteria of the Vermont Oxford Network Database were present: clinical signs of sepsis, positive blood culture for CONS, and intravenous antibacterial therapy for at least five days after obtaining blood culture or until death, in case it occurs within five days after obtaining blood culture [8]. Whenever CONS and another pathogen were identified in the same blood culture, only the other pathogen was recorded in the database, CONS was considered contaminant and this agent was discharged; age at the time of onset of sepsis considered to be the day on which the first blood culture for the event was positive: early-onset sepsis (EOS) defined as infection occurring before or at 72 hours of life and late-onset sepsis (LOS), after 72 hours of life [9]; organisms cultured and associated with death from sepsis; *Neonatal Comorbidities*: perinatal asphyxia defined as the presence of five-minute Apgar score below six and signs of encephalopathy as lethargy/stupor, hypotonia, and abnormal reflexes [10]; respiratory distress syndrome (RDS) defined as the need for oxigenotherapy, clinical features of RDS, and ventilation support and abnormal thorax radiograph in the first 24 hours of life [3]; necrotizing enterocolitis (NEC) classified according to the system of Bell et al. [11]; temperature instability, defined as temperature <36.5°C or >37.5°C or a variation of >1°C in a period of 24 hours and blood changes such as neutropenia, defined by a neutrophil count <1.5 × 10^3^/L and thrombocytopenia, defined as a platelet count <80 × 10^3^/L [11]. *Therapeutic Interventions*: use and length of use of intravascular catheter, defined as a peripherally inserted central catheter or by a vascular dissection and arterial or venous umbilical catheter, inserted before the onset of sepsis; use and length of use of mechanical ventilation and of total parenteral nutrition (TPN); time when enteral feeding was initiated and length of stay in the NICU. *Clinical Outcome*: neonatal death was considered related to infection when clinical signs and symptoms of sepsis evolved to signs and symptoms of irreversible septic shock as systemic hypotension, acidemia, anuria, and hypoxia; and/or signs and symptoms of irreversible disseminated intravascular coagulation syndrome as thrombocytopenia, major bleeding episodes as pulmonary hemorrhage, and, ultimately, progressing to death.

### 2.3. Statistical Analysis

Bivariate analysis was used to evaluate the association between potential risk factors and sepsis-related mortality. For statistical inference, the proportions were compared with the chi-square test (*χ* ^2^) or with Fisher's exact test with Yates correction when the expected value in any cell of a 2 × 2 table is <5. *P*-value of  .05 or less was considered significant. The cut-off for continuous data was obtained with the Receiver Operator Characteristics (ROC) curve. The cut-off was determined for the value with the highest accuracy from the area of the ROC curve, it means, to the highest area under the ROC curve. The odd ratios (ORs) were calculated with confidence intervals of 95% (CI95) for each risk factor. A stepwise backward unconditional multivariate logistic regression was performed to control for confounding and to assess the independence of the identified risk factors. All variables with a *P*-value of  .05 or less on bivariate analysis were included in the initial model. ORs and CI95 were calculated. The final model included the statistically significant variables in unconditional logistic regression analyzed by *P*-value of the test Walds. Statistical analysis was performed using the software Epi Info version 3.3.2, SPSS version 12, and Epi Data version 3.1.

The study was reviewed and approved by the local Research Ethics Committee (no. 000197).

## 3. Results

### 3.1. Incidence of Infection

Between 1 April, 2001 and 30 September, 2004, 203 VLBW infants were admitted to the NICU of SEH, RJ. During their stay in the NICU, 71/203 (35%) had one or more episodes of sepsis. For most VLBW infants, 61/71 (86%), sepsis onset occurred after 72 hours of life. Most of the VLBW infants, 55/71 (77.5%) had one episode; 11/71 (15.5%) two; 4/71 (5.6%) three; 1/71 (1.4%) four, totalling 87 episodes. A great number of the infected neonates, 30/71 (42.3%) were BW  ≤ 1000 g, classified as extreme low birth weight (ELBW) infants. The mean VLBW infant's age for all sepsis events was 12.9 (12.1) days, being 9.8 (9.3) for the first and 26 (15.6) for the second episode.

### 3.2. Pathogen Distribution

The majority of infection events, either EOS or LOS, were due to gram-positive organisms, 52/87 (59.8%). CONS was the most common pathogen of all events (31/87; 35.5%) and among gram-positive events (31/52; 59.6%). Gram-negative organisms account for 29/87 (33.3%) episodes, all LOS. *P. aeruginosa* was the most common gram-negative pathogen (8/87; 9.1%), followed by *Klebsiella sp*. and *Acinetobacter sp*. Yeasts were found in 6/87 (6.9%) episodes and *Candida albicans* was always the causing pathogen. The distribution of the organisms is shown in Table 1.

### 3.3. Antenatal, Intrapartum, and Neonatal History

Antenatal and intrapartum history, neonatal comorbidities, and therapeutic interventions were reviewed. The continuous data are presented as means and standard deviations and the categorical data are presented as percentages (Table 2).

### 3.4. Sepsis-Related Mortality

Overall 21 of the 71 VLBW infants with sepsis (29.6%) died, being 2/10 (20%) from EOS, and 19/61 (32.7%) from LOS. 

There were significant differences in death rates from sepsis, depending on the organisms isolated from the last positive culture (Table 3). Episodes of sepsis with gram-negative organisms were more likely to result in death than episodes with gram-positive or fungus (13/29; 44.8% versus 7/52; 13.4% versus 1/6; 16.7%; *P* < .05). Within this high-risk group, VLBW infants with *P. aeruginosa* sepsis were most likely to die (6/8; 75% of infants with *P. aeruginosa* infections died; *P* < .05).

ELBW infants (BW  ≤ 1000 g) were significantly more likely to die than those with BW  > 1000 g (17/30; 56.7% versus 4/41; 9.7%;  *P* < .05). The mean GA, BW, and five-minute Apgar score of VLBW infants who died were lower than that of those who survived (29.4 versus 32 weeks; 875 versus 1130 g; 7.4 versus 8.4; *P* < .05 each one).

VLBW infants submitted to intravascular catheter and mechanical ventilation were significantly more likely to die than those not submitted to these procedures (19/47; 40.4% versus 2/24; 8.3%; and 19/42; 45.26% versus 2/29; 6.9%; *P* < .05 each one). Enteral feeding onset before or at 72 hours of life and length of stay were highly significant predictors of survival (*P* < .05). The bivariate analysis is shown in Table 4.

### 3.5. Logistic Regression

The predictive model based on a stepwise backward unconditional multivariate logistic regression for 71 VLBW infants showed that BW independently contributed most to the dependent variable death, with a cut-off point of 1000 g. BW ≤1000 g infants presented a sepsis-related mortality rate 9.6 times higher than BW  > 1000 g infants (*P* < .05). The use of mechanical ventilation presented a clinically significant risk (sepsis-related mortality rate 6.7 times higher) but the statistical significance was marginal (*P* = .05). Length of stay was statistically significant with a negative coefficient, that is, the risk of death from sepsis decreased as length of stay increased (*P* < .05). The logistic regression analysis is shown in Table 5.

## 4. Discussion

In our four-year cohort, sepsis was frequent, 35%, with the highest risk of occurrence in the first two weeks of stay. Even thought was caused mainly by gram-positive organisms, represented by CONS, the mortality was higher when caused by gram-negative organisms, particularly *P. aeruginosa*. Neonatal sepsis-related mortality rate was high, 29.6%, in most cases involving ELBW infants, 56.7%, and due to gram-negative organisms, 44.8%, especially *P. aeruginosa*, 75%. The number of VLBW infants studied was low, however, representative of the whole population of neonates with BW  < 1500 g assisted in a NICU of a reference center for high-risk pregnant women, during the selected period. Being a retrospective study, there was a lack of comprehensive information about risk factors. For this reason, the possible risk factors of sepsis-related mortality were reported under Chart Review and included characteristics of the VLBW infants population and events related to infection episodes. 

There was a predominance of gram-positive organisms in EOS, especially CONS. This finding differs from the reports of Stoll et al. [9] and Rønnestad et al. [12] that, in the recent years, there was a change in the profile of the pathogens causing EOS in NICU, with increase of gram-negative organisms, especially *Escherichia coli*. The incidence of EOS due to *Group B Streptococcus* (GBS) was low, a single episode, probably because, in most cases the evaluated VLBW infants were product of the interruption of a high-risk pregnancy by caesarian section with ruptured of amniotic membranes at birth. According to Hickman et al. [13], these two factors contribute to the decrease of the vertical transmission of GBS and the lowest occurrence of EOS due to this agent, as in our study. 

There was a predominance of gram-positive organisms in LOS, 48.2%, mainly CONS, 27.5%. Costa et al. [14] in Portugal, Khashu [15] in Canada, Sarkar et al. [16] in the United State of America, and Richards et al. [17] in Colombia reported similar findings, what demonstrates that these organisms are prevalent agents of LOS in different countries and continents. *P. aeruginosa* was the most frequent agent of LOS, 9.1%, among gram-negative organisms. In contrast to our findings, Afroza [1] and Trotman and Bell [5] reported *K. pneumoniae* as the most usual infectious agent among gram-negative organisms in LOS. The reason for the high incidence of *P. aeruginosa* in our population must be object of future studies.

The sepsis-related mortality rate of VLBW infants was high, 29.6%, when compared to the rates reported in the literature, 17.3% to 21% [5, 6, 17]. VLBW infants with gram-negative sepsis were at the greatest risk of death, 44.8%. *P. aeruginosa* was the most aggressive agent of sepsis, responsible for the highest sepsis-related mortality rate, 75%, as reported by studies of Gordon and Isaacs [6].

BW  ≤ 1000 g was identified by logistic regression as the strongest independent sepsis-related mortality predictor. ELBW infants, a great vulnerable population to neonatal complications, presented the highest sepsis-related mortality rate, 56.6%. Afroza [1] related that, among many risk factors for infection, the single most important factor is low birth weight, as we observed. This finding suggests that efforts to reduce neonatal sepsis-related mortality must be turned especially to this population.

The use of mechanical ventilation was identified by logistic regression as a strong independent sepsis-related mortality predictor, which is in agreement with Flidel-Rimon et al. [18] and Stoll et al. [19]. This may be caused by the fact that neonates who need ventilation are the sickest patients, but also may be in part related to preventable ventilator-associated infection.

Enteral feeding initiated before or at 72 hours of life was a predictor of survival. Our findings suggest the practice of early onset of feeding, and of the discerning indication and maintenance of invasive procedures directed to the treatment of VLBW infants to control sepsis-related mortality in this population. 

In summary, sepsis-related mortality is very high in low birth weight infants, mainly in extreme low birth infants. This is particularly true for patients with positive blood cultures due to *P. aeruginosa*. Attention to the control of prematurity, knowledge of the neonatal flora and antibiotic sensitivity profile, introduction of early nutritional support with early onset enteral feeding and efforts to decrease ventilator related events may play a role in decreasing the mortality and morbidity of this very serious disease.

## Figures and Tables

**Table 1:** Number (%) of episodes of total sepsis, EOS and LOS in 71 VLBW infants testing positive for each organism, *Servidores Estado Hospital*, April 2001/September 2004.

ORGANISM	Total Sepsis		EOS		LOS	
	*N*	%	*n*	%	*n*	%
Gram-positive organisms	**52**	**59.8**	**10**	**11.6**	**42**	**48.2**
*Coagulase-negative Staphylococcus*	31	35.5	7	8	24	27.5
*Methicillin-sensible S. aureus*	16	18.3	1	1.2	15	17.1
*Methicillin-resistant S. aureus*	1	1.2	—	—	1	1.2
*Enterococcus*	2	2.4	—	—	2	2.4
*Streptococcus agalactiae (B)*	1	1.2	1	1.2	—	—
*Listeria monocytogenes*	1	1.2	1	1.2	—	—
Gram-negative organisms	**29**	**33.3**	—	—	**29**	**33.3**
*Pseudomonas aeruginosa*	8	9.1	—	—	8	9.1
*Klebsiella pneumoniae sp*	6	6.9	—	—	6	6.9
*Acinetobacter calcoaceticus*	6	6.9	—	—	6	6.9
*Unidentified gram-negative*	3	3.4	—	—	3	3.4
*Serratia marcescens*	3	3.4	—	—	3	3.4
*Enterobacter cloacae*	2	2.4	—	—	2	2.4
*Escherichia coli*	1	1.2	—	—	1	1.2
Fungi	**6**	**6.9**	—	—	**6**	**6.9**
*Candida albicans*	6	6.9	—	—	6	6.9

Total	**87**	**100**	**10**	**11.6**	**77**	**88.4**

EOS: early-onset-sepsis.

LOS: late-onset-sepsis.

**Table 2:** Risk factors for sepsis-related mortality in 71 VLBW infants with sepsis, *Servidores EstadoHospital*, April 2001/September 2004.

Risk factors: antenatal and intrapartum history,		
neonatal comorbidities, therapeutic interventions		
*Continuous variables*	*n*	%

Rupture of membranes at birth	55	77.5
<12 hours before birth	3	4.2
Caesarian section	53	74.6
Males	36	50.7
Birth weight ≤ 1000 g	30	42.3
Small for gestational age	45	63.4
Perinatal asphyxia	37	52.1
Respiratory distress syndrome	39	54.9
Necrotizing enterocolitis	12	16.9
Temperature instability	12	16.9
Neutropenia	14	19.7
Thrombocytopenia	44	61.9
Intravascular catheter	47	66.2
Total Parenteral nutrition	64	90.1
Mechanical ventilation	42	59.2
Enteral feeding onset > 72 hours	18	25.3

*Categorical variables*	Mean	Standard deviation

Gestational age (weeks)	31	2.5
1-minute Apgar score	6.2	2.1
5-minute Apgar score	8.1	1.4
10-minute Apgar score	7.2	2
Birth weight (grams)	1054	232
Lenght of intravascular catheter (days)	10.37	8.7
Lenght of total parenteral nutrition (days)	9.12	7.52
Lenght of mechanical ventilation (days)	6.97	9.46
Length of stay (days)	53.8	35

**Table 3:** Number (%) of organisms related to total sepsis, EOS and LOS in 21VLBW infants who died of sepsis, *Servidores do Estado Hospital*, April 2001/September 2004.

	Sepsis-related mortality						
Organism	Total sepsis			EOS		LOS	
	**N**	**n**	%	**n**	%	*n*	%
Gram-positive organism	**52**	**7**	**13.4**	**1**	**1.9**	**6**	**11.5**
*Coagulase-negative staphylococcus *	31	4	12.9	1	3.2	3	9.6
*Staphylococcus aureus*	16	2	12.9	–—	–—	2	12.5
*Staphylococcus MRSA*	1	1	100	–—	–—	1	100
Others	4	–—	–—	–—	–—	–—	–—
Gram-negative organism	29	**13**	**44.8**	**1**	**3.4**	**12**	**41.4**
*Pseudomonas aeruginosa*	8	6	75		–—	6	75
*Klebsiella pneumoniae sp*	6	4	66.7	–—	–—	4	66.7
*Serratia*	3	2	66.7	1	33.3	1	33.3
*Escherichia coli*	1	1	100			1	100
Others	11	–—	–—	–—	–—	–—	–—
Fungi	6	**1**	**16.7**	**–—**	**–—**	**1**	**16.7**
*Candida albicans*	6	1	16.7	–—	–—	1	16.7

Total	87*	21**		2		19	

*Total episodes sepsis-causing organisms

**Organisms that caused the last sepsis episode right before death of the 21 VLBW infants

EOS: early-onset-sepsis.

LOS: late-onset-sepsis.

**Table 4:** Risk factors associated with sepsis-related mortality and sepsis relate survival of 71 VLBW infants with sepsis,* Servidores Estado Hospital,* April 2001/September 2004.

Risk factor	OR	CI 95	*P*
*Sepsis-related mortality*			
Gestational age ≤ 28 weeks	14.67	2.12–116.07	.000
Birth weight ≤ 1000 g	10.93	2.76–47.15	.000
5th minute Apgar ≤ 7	5.41	0.75–47.61	.064 (Fisher)
Gram-negative organisms	2.91	1.14–7.45	.023
Use of mechanical ventilation	11.05	2.34–53.04	.003
Use of intravascular catheter	3.11	1.08–8.93	.003
*Sepsis-related survival*			
Enteral feeding onset ≤ 72 hours	0.10	0.00–0.79	.022

OR = odds ratio

IC 95 = 95% confidence interval.

**Table 5:** Risk factors for mortality and for survival in the 71VLBW infants with sepsis, identified by predictive model based on a backward step-by-step unconditional multivariate logistic regression.

	Risk Factor	OR	CI95	*P*
Step 1(a)	Length of stay	0.933	0.900–0.967	.000
Step 2(b)	Length of stay >33 days	0.938	0.905–0.972	.000
	Birth weight ≤1000 g	13.232	2.603–67.249	.002
Step 3(c)	Mechanical ventilation	6.726	1.043–43.363	.045
	Length of stay >28 days	0.944	0.912–0.978	.001
	Birth weight ≤1000 g	9.600	1.702–54.156	.010

Odds ratio (OR) for the probability of death by sepsis

IC95 = 95% confidence interval.

(a) variable used in step  1 length of stay >28 days

(b) variable used in step  2 birth weight ≤1000 g.

(c) variable used in step  3 mechanical ventilation

*R* ^2^ = 0.685.

## References

[1] Afroza S (2006). Neonatal sepsis—a global problemml: an overview. *Mymensingh Medical Journal*.

[2] Görbe E, Jeager J, Nagy B (2007). Assessment of serum interleukin-6 with a rapid test. The diagnosis of neonatal sepsis can be established or ruled out. *Orvosi Hetilap*.

[3] Makhoul IR, Sujov P, Smolkin T, Lusky A, Reichman B (2005). Pathogen-specific early mortality in very low birth weight infants with late-onset sepsis: a national survey. *Clinical Infectious Diseases*.

[4] Sohn AH, Garrett DO, Sinkowitz-Cochran RL (2001). Prevalence of nosocomial infections in neonatal intensive care unit patients: results from the first national point-prevalence survey. *Journal of Pediatrics*.

[5] Trotman H, Bell Y (2006). Neonatal sepsis in very low birthweight infants at the University Hospital of the West Indies. *West Indian Medical Journal*.

[6] Gordon A, Isaacs D (2006). Late onset neonatal gram-negative bacillary infection in Australia and New Zealand: 1992–2002. *Pediatric Infectious Disease Journal*.

[7] Usher R, McLean F (1969). Intrauterine growth of live-born Caucasian infants at sea level: standards obtained from measurements in 7 dimensions of infants born between 25 and 44 weeks. *The Journal of Pediatrics*.

[8] (1993). *Vermont Oxford Network Database Manual of Operations, Release 2.0*.

[9] Stoll BJ, Hansen NI, Higgins RD (2005). Very low birth weight preterm infants with early onset neonatal sepsis: the predominance of Gram-negative infections continues in the National Institute of Child Health and Human Development Neonatal Research Network, 2002-2003. *Pediatric Infectious Disease Journal*.

[10] Battin MR, Penrice J, Gunn TR, Gunn AJ (2003). Treatment of term infants with head cooling and mild systemic hypothermia (35.0°C and 34.5°C) after perinatal asphyxia. *Pediatrics*.

[11] Bell MJ, Ternberg JL, Feigin RD (1978). Neonatal necrotizing enterocolitis: therapeutic decisions based upon clinical staging. *Annals of Surgery*.

[12] Rønnestad A, Tore G, Sverre AM (2005). Septicemia in the first week of life in a Norwegian national cohort of extremely premature infants. *Pediatrics*.

[13] Hickman ME, Rench MA, Ferrieri P, Baker CJ (1999). Changing epidemiology of group B streptococcal colonization. *Pediatrics*.

[14] Costa A, Guimaraes H, Souto A (1996). Sepsis in new-borns with very low birth weight. *Acta Medica Portuguesa*.

[15] Khashu M, Osiovich H, Henry D, Al Khotani A, Solimano A, Speert DP (2006). Persistent bacteremia and severe thrombocytopenia caused by *coagulase-negative Staphylococcus* in a neonatal intensive care unit. *Pediatrics*.

[16] Sarkar S, Bhagat I, Hieber S, Donn SM (2006). Can neutrophil responses in very low birth weight infants predict the organisms responsible for late-onset bacterial or fungal sepsis?. *Journal of Perinatology*.

[17] Richards C, Alonso-Echanove J, Caicedo Y, Jarvis WR (2004). *Klebsiella pneumoniae* bloodstream infections among neonates in a high-risk nursery in Cali, Colombia. *Infection Control and Hospital Epidemiology*.

[18] Flidel-Rimon O, Friedman S, Lev E, Juster-Reicher A, Amitay M, Shinwell ES (2004). Early onset enteral feeding and nosocomial sepsis in very low birth weight infants. *Archives of Disease in Childhood*.

[19] Stoll BJ, Hansen N, Fanaroff AA (2002). Late-onset sepsis in very low birth weight neonates: the experience of the NICHD Neonatal Research Network. *Pediatrics*.

